# Siglecs Modulate Activities of Immune Cells Through Positive and Negative Regulation of ROS Generation

**DOI:** 10.3389/fimmu.2021.758588

**Published:** 2021-11-03

**Authors:** Joyshree Karmakar, Kaustuv Mukherjee, Chitra Mandal

**Affiliations:** Cancer Biology and Inflammatory Disorder Division, Council of Scientific and Industrial Research (CSIR)-Indian Institute of Chemical Biology, Kolkata, India

**Keywords:** chronic obstructive pulmonary disease, coronavirus disease (COVID-19), *Leishmania donovani*, neutrophil, reactive oxygen species, siglecs, Siglec-sialoglycan, eosinophil

## Abstract

Reactive oxygen species (ROS) are a group of oxygen-containing highly-reactive molecules produced from oxidative metabolic processes or in response to intracellular signals like cytokines and external stimuli like pathogen attack. They regulate a range of physiological processes and are involved in innate immune responses against infectious agents. Deregulation of ROS contributes to a plethora of disease conditions. Sialic acids are carbohydrates, present on cell surfaces or soluble proteins. Sialic acid-binding immunoglobulin-like lectins (Siglecs) recognize and bind to sialic acids. These are widely expressed on various types of immune cells. Siglecs modulate immune activation and can promote or inhibit ROS generation under different contexts. Siglecs promote ROS-dependent cell death in neutrophils and eosinophils while limiting oxidative stress associated with chronic obstructive pulmonary disease (COPD), sickle cell disease (SCD), coronavirus disease-2019 (COVID-19), etc. This review distinguishes itself in summarizing the current understanding of the role of Siglecs in moderating ROS production and their distinct effect on different immune cells; that ultimately determine the cellular response and the disease outcome. This is an important field of investigation having scope for both expansion and medical importance.

## Introduction

Sialic acids (SA), a family of nine-carbon acidic monosaccharides, are commonly found at terminal positions in glycan chains attached to glycoproteins/glycolipids (sialoglycans) present on cell surfaces or soluble proteins. So far, nearly 50 different derivatives of SA have been discovered (1). Sialic acids are commonly found in higher mammals but also in several protozoans, bacteria, and fungi (1). Sialic acid-binding immunoglobulin-like lectins (Siglecs) are a class of glycan-binding proteins that recognize and bind to these SA (2–4). Several Siglecs modulate cellular activity and response *via* signaling through cytoplasmic regulatory motifs.

Reactive oxygen species (ROS) refers to oxygen-derived, highly reactive molecules which include superoxide anion and hydrogen peroxide (5). ROS are produced in mitochondria as by-products of the electron transfer chain during aerobic respiration. Phagocytic cells (macrophages, neutrophils, and dendritic cells) also generate ROS and reactive nitrogen species (RNS) as components of “oxidative burst” for degrading biomolecules and internalized pathogens (6).

In this review, we have attempted to highlight how Siglec-sialoglycan interactions modulate ROS generation in various immune cells depending on physiological states or infections.

### Distribution and Classification of Siglecs

Most immune cells of hematopoietic origin express one or more kinds of Siglecs (7). Although resting T cells show low Siglec expression, a few T cell subsets express Siglecs (particularly Siglec-5, 7, 9, and 10) after activation or in specific contexts (8–10) (**Table 1**). Some Siglecs, like Siglec-9, are expressed on several immune cells. However, expression of certain Siglecs is restricted to particular cell types; like Siglec-1 (Sialoadhesin) on monocytes, macrophages, and dendritic cells (15). Siglec-2 (CD22) is predominantly observed on B cells though it is also expressed at low levels on mast cells, dendritic cells, and basophils (17–20) (**Table 1**).

**Table 1:** Distribution of human and mice siglec families on different cells, ligand preferences and their common functions.

Siglecs	Distribution on cells	Linkage preference on sialylated ligands	Functions	References
**Evolutionary conserved siglecs**				
Siglec-1 (CD-169)	Macrophages	α2,3	Recognition and phagocytosis of sialylated pathogens. Modulates immune response through cell-cell interactions	(7, 11–15)
Siglec-2 (CD22)	Predominantly on B-cells, also detected in Dendritic cells Mast cell Basophils, Gut eosinophils of mouse	α2,6- preferably, Neu5Gc/ Neu5Ac	Regulates B cell survival, signaling and homeostasis	(7, 11, 12, 14, 16–20)
Siglec-4 (MAG)	Neuronal cells	α2,3**>** α2,6	Secures myelin-axon associations through binding with axonic gangliosides GD1a and GT1b. Regulates axon growth and survival	(7, 12)
Siglec-15	Osteoclasts, macrophages	α2,6	Regulates osteoclast differentiation, involved in regulation of immune response	(11–13, 16)
**Siglec-3 (CD-33) related siglecs**				
Siglec-3	Myeloid progenitors	α2,6**>** α2,3	Involved in immune inhibitory functions	(7, 11, 12)
Siglec-5	Monocytes, neutrophils, activated T cells	α2,3	Involved in pathogen phagocytosis and clearance of sialylated substrates	(7, 9, 10, 12)
Siglec-6	Trophoblasts, Mast cells B cells	α2,6- is preferred	Highest expression levels in placenta but function in gestation still not explored	(11, 12)
Siglec-7	Neutrophils, monocytes, mast cells, NK cells, CD8-T cell subset	α2,8- is preferred	Inhibitory in nature, down regulates T cell signaling	(11, 12)
Siglec-8 (Human)	Eosinophils, basophils, mast cells	α2,3- and sulphated ligands	Involved in cellular apoptosis	(7, 12)
Siglec-F (closely related functional murine paralog)	Eosinophils Macrophages			
Siglec-9 (Human)	Neutrophils, monocytes, NK cells, dendritic cells, B cells, CD8-T cell subset	α2,3 or α2,6- or sulfated ligands	Involved in cellular response apoptosis and inhibition of immune response	(7, 9, 10, 12)
Siglec-E (murine paralog)	Neutrophils Monocytes dendritic cells			
Siglec-10 (Human)	NK cells, B cells, monocytes eosinophils, T cells	α2,3 or α2,6	Inhibits calcium signalling mediated by B cell receptor	(7, 10, 11)
Siglec-G (Murine paralog)	B cells, dendritic cells			
Siglec-11	Macrophage, B cells, microglia, ovary stroma	α2,8- is preferred	Involved in pathology of neurodegenerative diseases	(7, 11)
Siglec-14 (Human)	Monocytes, neutrophils	α2,3	Functions as an activating receptor through association with DNAX Activating Protein of 12 kDa (DAP12)	(7, 14)
Siglec-H (Murine paralog)	Monocytes neutrophils Microglia			
Siglec-16	Microglia	α2,8- is preferred	Involved in Pathology of neurodegenerative disease	(7, 11)

This table has been compiled from information collected from references # (7, 9–20). The distribution of siglecs on human and murine immune cells is indicated here.

Siglecs are classified into two major groups based on sequence homology. The first group (Siglec-1, CD22, Siglec-4, and Siglec-15) has low sequence similarity between each other but is conserved across species. In contrast, CD33-related Siglecs (CD33r Siglecs) are closely related but not highly conserved (21). Siglecs have been extensively studied in humans, mice, and few other animals. Humans express around 11 different CD33r Siglecs (Siglec-3,-5,-6,-7,-8,-9,-10,−11,−14,−16,−17) while mice express only a few (Siglec-3,-E,-F,-G,-H) (11). Therefore, murine equivalents of only a few human Siglecs are known (**Table 1**).

### Siglec Affinities and Ligand Preferences

Sialic acids are attached to glycans *via* α-glycosidic linkages formed between its C2 with C3 (α2,3) or C6 (α2,6) positions of galactose or C6 position of N-acetylgalactosamine. Polymers of SA are termed polysialic acid, where successive SA residues are primarily linked *via* α2,8 linkages, or by α2,9 linkages in a few cases (1). Different Siglecs have characteristic affinities towards specific sialoglycans based on the SA position, linkages, and surrounding sugars (**Table 1**). Siglec-9 recognizes SA linked to galactose *via* α2,3/α2,6 linkages and other sialylated structures like sialyl Lewis-x (SLex), 6-sulfo-SLex (22, 23). However, Siglec-8 prefers α2,3 linked SA attached to sulfated galactose, as seen in 6-sulfo-SLex (22). Ligand preferences of several Siglecs have been elucidated through glycan-binding arrays, molecular modeling studies, cell-based binding assays, etc. (22). Siglecs can bind sialoglycans present on the same cell (*cis*-interactions) or extracellular ligands present on neighboring cells or secretory glycoproteins (*trans*-interactions).

### Siglec Clustering Enhances Their Signaling Activity

**Figure 1:**
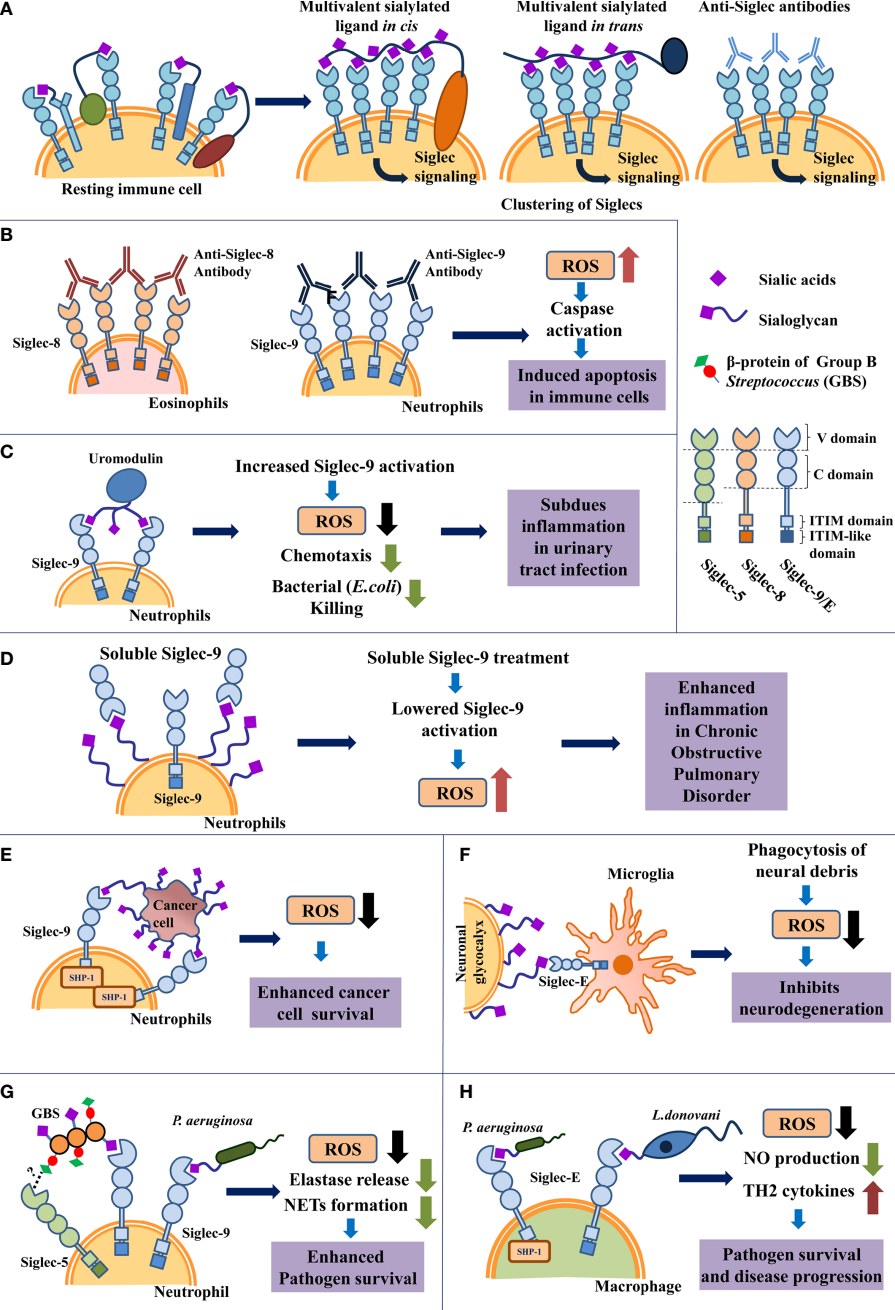
Schematic representation of ROS modulation by Siglecs in immune cells under different pathological conditions. **(A)** Schematic representation of clustering of Siglecs on immune cell surface induced by interaction with various multivalent ligands. **(B)**. Siglec-crosslinking using anti-Siglec antibodies promote ROS generation which triggers cellular apoptosis in resting neutrophils and eosinophils. **(C)** Uromodulin binding leads to enhanced Siglec-9 activation in neutrophils *in vitro*, inhibiting ROS production. This interaction possibly limits excessive inflammation during urinary tract infection. **(D)** Increased expression of soluble Siglec-9 (sSiglec-9) lowers Siglec-9-sialic acids engagement on neutrophils, leading to increased inflammation and ROS generation in chronic obstructive pulmonary disease (COPD). **(E)** Cancer cell sialoglycoproteins engage Siglecs on neutrophil *via* Siglec-sialoglycan interaction to reduce ROS production, which enhances cancer cell survival. **(F)** Siglec-E engagement with neural sialoglycoproteins suppresses ROS generation by microglial cells and prevents oxidative stress mediated neuro-degeneration. **(G)** Sialylated bacteria like Group B *Streptococcus* (GBS) and *Pseudomonas aeruginosa* interact with Siglec-9 present on neutrophil using sialic acids present on their surface. Additionally, non-sialylated GBS β-protein of GBS also binds with Siglec-5 present on neutrophil in a sialic acid-independent manner. Both Siglec-sialoglycan and Siglec-protein interactions encourage pathogen survival through subdued immune response. However, the exact binding site of protein mediated interaction between Siglec-5 and GBS β-protein, remains unknown. **(H)** Interaction of macrophage Siglec-E with sialylated pathogens like *Pseudomonas aeruginosa* and *Leishmania donovani* respectively subdues ROS production for disease progression.

Siglec-sialoglycan binding is weak and transient (24). Interaction between Siglecs with multivalent ligands leads to Siglec clustering, which increases the strength of Siglec-ligand binding and initiates cellular signaling (22, 25–27). Multivalent ligands present several Siglec-binding sites. These may be extracellular ligands, antibodies, synthetic agonists interacting with Siglecs in *trans*, or cell surface sialoglycans, membrane-bound synthetic ligands binding in *cis* (26, 27). Siglec-clustering into nanodomains was revealed by high-resolution microscopy (28, 29). Siglec clustering into signaling domains by anti-Siglec antibodies is shown (**Figure 1A**).

### Function

Siglecs are transmembrane proteins that generally interact with sialoglycans through the carbohydrate recognition domain present in their extracellular *N*-terminal immunoglobulin-like folds (V-set domain). Several Siglecs contain immunoreceptor tyrosine-based inhibitory motifs (ITIMs) in their cytoplasmic domains while some are associated with adaptor proteins that contain immunoreceptor tyrosine-based activatory motifs (ITAMs) (7, 30). Signaling *via* ITIM leads to recruitment of Src homology region domain-containing phosphatase-1 and 2 (SHP-1, SHP-2) which inhibits phosphorylation-based cellular signaling pathways (14, 31). Siglec-sialoglycan binding leads to modulation of cellular activity due to signaling *via* either ITIMs (inhibitory) or ITAMs (activatory) motifs (21).

Siglecs play several roles in normal physiology, including immunomodulation, phagocytosis of sialylated pathogens (mainly Siglec-1); regulation of B cell signaling and survival (CD22); maintenance of axon-myelin interactions, axon growth (Siglec-4); regulation of osteoclast differentiation (Siglec-15), etc (12–14, 16). Distribution, ligand preference, and functions of a few human Siglecs have been compiled in **Table 1**.

### Effect of ROS

ROS has a direct detrimental effect on lipids, proteins, and DNA. However, low levels of ROS are required for maintaining metabolism, signal transduction, cell proliferation, apoptosis, and aging process (32). Oxidative stress due to excessive ROS generation is implicated in asthma, chronic obstructive pulmonary disease (COPD), diabetes, cardiovascular diseases, auto-immunity, and neurodegenerative diseases (5). Phagocytic cells express a multi-subunit NADPH-dependent phagocytic oxidase (Phox or NOX2), which produces ROS as a component of antimicrobial defense (33). Some components of NOX2 (p67^phox^, p47^phox^, p40^phox^, a small G protein-rac1, rac2), are localized in the cytosol and some are membrane-associated (gp91^phox^, p22^phox^). The activity of membrane-associated gp91^phox^ (34) is indispensable for NOX2 function, which uses NADPH as an electron donor to generate superoxide anion (O_2_^*-^) by oxygen reduction (35–37).

### Siglec-Based Modulation of ROS Production

Siglecs modulate ROS production through Siglec-sialoglycan binding, signaling through ITIMs or direct protein-protein interactions. Siglec-9 was identified as a potential ligand for human amine oxidase type 3 (hAOC3) by phage peptide library-based screening (38). Flow cytometry confirmed the binding between recombinant Chinese Hamster Ovary cells (CHO) expressing hAOC3 and CHO-expressing Siglec-9 (39). Surface plasmon resonance (SPR) based binding assays demonstrated that Siglec-9 C2_2_ domain binds to the active site of hAOC3. Introduction of point mutations followed by SPR-based binding assays identified Arg284 and Arg290 in Siglec-9 to be critical for its binding with hAOC3. Such Siglec-binding with oxidase enzyme through protein-protein interactions enhances hAOC3 activity and generates hydrogen peroxide (39). Siglec-9 also binds to sialoglycoprotein hAOC3 through sialic acid-Siglec binding *via* its V domain (40).

Siglec-E knockout neutrophils infected with non-sialylated *Escherichia coli* strains (Gram-negative bacteria) produced significantly less amount of ROS than wild-type neutrophils, suggesting that Siglec-E promoted ROS generation (41). Silencing Siglec-E or Siglec-9 (the human equivalent of Siglec-E) also reduced ROS generation in neutrophils/THP-1 cells after *E.coli* infection (41). Immunoprecipitation revealed that endogenous Siglec-E associates with NOX2 subunits (gp91^phox^/p47^phox^ *)* in *E.coli*-infected murine neutrophil and Tyr432 residue was found to be critical for Siglec-E-p47^phox^ binding. Cells overexpressing Siglec-E with mutated Tyr432 also produced lower amounts of ROS following *E.coli* infection. This confirmed that Tyr432 in the ITIM domain of Siglec-E is needed for its association with NOX2 subunit p47^phox^ which promotes ROS production (41).

Interestingly, treatment of primary human neutrophils with a synthetic Siglec-9 agonist (pS9L) or anti-Siglec-9 antibody also leads to ROS generation which is inhibited upon SHP-1/2 inhibitor treatment. This indicates that Siglec-9 engagement also promotes ROS generation through SHP-1/SHP-2 signaling (42).

### Siglec Engagement on Immune Cells Modulates ROS Generation

#### Eosinophils

Treating resting eosinophils with anti-Siglec-8 monoclonal antibodies and secondary polyclonal antibodies leads to extensive crosslinking or clustering of Siglec-8 (**Figure 1B**, left panel). Siglec-8 clustering was followed by ROS generation, reduction in mitochondrial membrane potential, and cleavage of caspases; culminating in cellular apoptosis (43, 44). Eosinophils incubated with pro-survival cytokine IL-5 show further increased cell death upon Siglec-8 cross-linking (45). These IL-5-activated eosinophils exhibit caspase-independent necrotic death, involving ROS generation and increased phosphorylation of MEK1, ERK1/2 (46). Treatment with ROS inhibitors confirmed that Siglec-crosslinking-mediated ROS production is essential for triggering eosinophil death in both resting and activated cells. Produced ROS was accumulated intracellularly in eosinophils (47).

In contrast, eosinophils stimulated with pro-survival cytokine IL-33 also exhibit enhanced cell death after Siglec-8 cross-linking, but without any significant increase in ROS generation (48). The lack of ROS production in IL-33-stimulated eosinophils remains to be explored.

Siglec-F shows a similar ligand binding profile like Siglec-8 and is also expressed on eosinophils. It is considered as a functionally convergent paralog of Siglec-8 in mice (49, 50). Mice treated with anti-Siglec-F antibodies show induction of caspase-dependent eosinophil death independent of NADPH oxidase activity and ROS production (51).

Binding Siglec-8 with monoclonal antibodies or synthetic Siglec-8 ligands leads to increased expression of CD11b/CD18. CD11b/CD18 heterodimer belongs to the β2-integrin subgroup, which increases the adhesiveness of IL-5-stimulated eosinophils. Such Siglec-8 engagement also leads to a time-dependent increase in ROS production, dependent on β2-integrin expression (52). NADPH oxidase (NOX) enzyme was identified as the source of ROS (52). This indicates antibodies or ligand-based Siglec-8 binding triggers ROS production through probable modulation of NOX activity.

#### Neutrophils

Neutrophils are the most abundant type of immune cells present in blood. Neutrophils perform immunosurveillance and respond to infiltrating microbes by phagocytosis, respiratory burst, degranulation, and formation of neutrophil extracellular traps (NETs) (53). Following successful clearance of microbes, responding neutrophils generally undergo apoptosis. However, pro-survival cytokines or growth factors in the milieu often prevent such apoptosis, leading to chronic or acute inflammations.

Antibody-based cross-linking of Siglec-9 triggers apoptosis in normal resting neutrophils due to ROS generation and caspase cleavage (**Figure 1B**, right panel) (54). Interestingly, neutrophils isolated from patients suffering from inflammatory conditions like rheumatic arthritis, acute septic shock, etc. show enhanced expression of Siglec-9 and increased death upon Siglec-9 cross-linking. Neutrophils stimulated with GM-CSF, interferon-α or interferon-γ (IFN-α/γ) similarly exhibit increased cell death after Siglec-9 cross-linking (47).

Such cytokine-stimulated neutrophils mainly showed non-apoptotic cell death upon Siglec-binding, characterized by cytoplasmic vacuolization, non-involvement of caspases, and only ROS generation. However, ROS generated by NADPH oxidase was identified to be crucial in both kinds of cell death (54).

Neutrophils express adhesion factors (like CD11b containing β2-integrins) for attachment and migration from blood to other sites of injury or infection. They adhere to fibrinogen-coated plates *via* CD11b-fibrinogen binding, leading to integrin-triggered ROS production (55). Additionally, Siglec-E binding with the sialoglycoprotein fibrinogen further increases ROS generation *via* NADPH-oxidase activity, which required fibrinogen-CD11b binding. In summary, Siglec-8 in eosinophils (52) and Siglec-9/E in neutrophils (54, 55) modulate NOX activity for promoting ROS generation.

Additionally, Siglec-E-mediated β2-integrin-dependent ROS generation prevented migration of neutrophils into the lungs of mice exposed to lipopolysaccharide, which was reversed upon inhibition of NOX activity (55).

#### In Inflammation

In normal physiology, Siglec-sialoglycan mediated signaling modulates immune cell activation, their response, ROS release, etc for preventing excessive inflammation and tissue injury (6).

#### Urinary Tract Infections (UTI)

Tamm-Horsfall protein (THP, uromodulin), is the most abundant protein in urine. It is a sialoglycoprotein that binds to Siglec-9/Siglec-E. Uromodulin treatment of neutrophils leads to reduced ROS generation, lowered chemotaxis, and reduced killing of uropathogenic *Escherichia coli* (56). During urinary tract infections, Uromodulin-Siglec-9 interaction possibly limits excessive neutrophil infiltration, reduces ROS generation, thereby modulating tissue inflammation (**Figure 1C**).

#### Chronic Obstructive Pulmonary Disorder (COPD)

Siglec-based regulation of ROS levels is observed in COPD patients. These patients exhibit elevated levels of the extracellular domain of Siglec-9 (soluble Siglec-9/sSiglec-9) in their plasma along with neutrophil hyperactivation, chemotaxis, and increased oxidative stress (57) (**Figure 1D**). Healthy neutrophils treated with sSiglec-9 *in vitro* show lower binding between neutrophil surface Siglec-9 and sialoglycans and thereby reducing Siglec-9 activation. This leads to increased ROS production and chemotaxis by neutrophils (57). Expression of Siglec-9 was upregulated in peripheral blood and alveolar neutrophils in COPD patients, possibly to enhance Siglec-based inhibitory signaling in response to neutrophil hyperactivation (57).

#### Sickle Cell Disease (SCD)

Siglec-based regulation of neutrophil activation is also seen in sickle cell disease (SCD). Neutrophils cultured with healthy erythrocytes showed lowered activation and ROS generation. Healthy erythrocytes suppress neutrophil activation by engaging with neutrophil Siglec-9 *via* sialylated erythrocyte membrane proteins like glycophorin A (58). In SCD, erythrocytes rapidly age, losing membrane elasticity and undergoing changes in cell membrane composition and protein expression. SCD-erythrocytes contain more SA compared to healthy erythrocytes but show lowered binding with neutrophil-Siglec-9 (59). Consequently, culturing healthy neutrophils with SCD erythrocytes leads to increased ROS release due to lowered Siglec-9 activation. Hyperactivation of neutrophils may lead to systemic inflammation, vaso-occlusion, etc. commonly observed in SCD.

#### Cancer Progression

Several types of cancers exhibit increased sialylation (60, 61). Neutrophils incubated with cancerous cells *in vitro* showed enhanced Siglec-9 engagement with cancer cell sialoglycoproteins, which increased SHP-1 recruitment. Consequently, ROS production and killing of tumor cell was inhibited (62) (**Figure 1E**). Mice lacking Siglec-E (murine equivalent of Siglec-9) show increased neutrophil activity, immunosurveillance, and killing of injected tumor cells (62).

Surprisingly, established tumors grew quicker in mice lacking Siglec-E. Tumor-infiltrating macrophages in such mice showed an M2 type phenotype. Depending on environmental stimuli, macrophages are activated into M1 (pro-inflammatory, eliminates tumor cells) or M2 (promotes cell proliferation, wound repair) macrophages (63). Peritoneal macrophages from mice lacking Siglec-E showed upregulation of M2 polarization markers upon co-culture with cancerous cells. Therefore, depending on the stage of cancer progression, the absence of Siglec-E can enhance cancer cell killing (by neutrophil activity like ROS generation) or promote tumor growth (by inducing macrophage M2 polarization).

#### Coronavirus Infections

During the COVID-19 pandemic, coronavirus infections have been linked with uncontrolled inflammatory responses, hyperactivation of neutrophils, and NETs formation (64). Incubation with serum/plasma from COVID-19 patients triggered NETosis in neutrophils from healthy donors (65, 66). Siglec-9 engagement inhibits neutrophil activity and induces apoptosis. A Siglec-9 agonist, a glycopolypeptide bearing modified SA residues and lipid moieties (pS9L), induces Siglec-9 clustering through *cis* interactions in macrophages after membrane insertion (28). Incubation of neutrophils with this agonist leads to ROS generation *via* SHP-1/2 and blocked NETosis induced by COVID-19 plasma or TLR 7/9 agonists (42). This agonist may reduce inflammation by blocking NETosis and by triggering neutrophil apoptosis through ROS generation (54).

#### Polysialic Acid-Based Nanoparticles

Polysialic acids serve as multivalent ligands and activate Siglecs. Treatment of neutrophils with phorbol 12-myristate 13-acetate (PMA) induces NETosis and ROS generation. However, incubating neutrophils with aliphatic amine latex nanoparticles coupled to polysialic acids having <9 sialic acid residues, leads to a reduction in PMA-induced ROS levels and NETs formation (67). These polysialylated nanoparticles possibly function as ligands for neutrophil surface Siglec-5. Similarly, injection of polysialic acids having ~20 SA residues in transgenic mice prevented ROS generation by Siglec-11-expressing phagocytes. Age-related macular degeneration happens due to excessive ROS production, complement deposition which may be prevented by targeting siglec-11 *via* polysialic acids (68).

All these studies suggest that enhancing Siglec-sialoglycan engagement may be beneficial for controlling ROS levels in inflammatory disorders.

#### Neurodegenerative Diseases

Microglia are immune cells resident in the central nervous system, responsible for detecting invasive pathogens and maintaining tissue homeostasis by removing damaged, apoptotic or unnecessary synapses, neurons, and plaques. Microglial cells produce ROS following the phagocytosis of apoptotic bodies. However, oxidative stress in the central nervous system leads to neurodegenerative problems. SA content is highest in mammalian brains. Silencing Siglec-E expression in microglia confirmed that microglial Siglec-E binds to the sialylated neuronal glycocalyx to suppress ROS release following phagocytosis of neuronal debris (69). Here, Siglec-E regulates microglial ROS to prevent neurodegeneration (**Figure 1F**).

#### Early Aging

The lifespan of mammals is positively correlated with the number of CD33r Siglec genes (70). Siglecs are responsible for regulating ROS, particularly produced by activated phagocytes *via* NOX enzyme, during inflammatory responses. ROS possibly plays a role in early aging (71). Deletion of Siglec-E in mice resulted in shortened lifespan with increased generation of ROS and inhibition of ROS-detoxification systems (70). This correlation was confirmed across 26 species and held true for both activatory and inhibitory Siglecs (72).

Although a positive correlation exists between mammalian lifespan and siglecs, further research is needed to clearly understand how both the activatory and inhibitory siglecs are able to regulate the specific signaling pathways and receptors involved in neurodegenerative diseases.

#### Down-Regulation of ROS in the Infection Process Through Siglec-Sialoglycan Interaction

The capsular cell wall on Group B *Streptococcus* (GBS) is sialylated and associated with its virulence (73–77). These sialoglycans interact with Siglec-9 on human neutrophils for suppressing neutrophil oxidative burst and NETs formation, thus leading to enhanced bacterial survival (**Figure 1G**). A similar interaction was also reported with Siglec-E expressed on murine macrophages (76, 78).

Additionally, Group A *Streptococcus* expresses high molecular weight hyaluronan (HMW-HA). A similar sialylated molecule is also expressed on human neutrophils. Thus, Group A *Streptococcus* activates Siglec-9 on neutrophils through molecular mimicry to subdue ROS production, decrease NETs and apoptosis, for securing its existence inside the host (76, 79).

A few GBS strains express a non-sialylated β-protein docked on their cell wall that can efficiently engage Siglec-5 (**Figure 1G**). Such interaction was confirmed through the binding of SA-deficient mutant GBS with hSiglec-5-Fc. Trypsin treatment prevents this interaction by degrading the β**-**protein on the pathogen, indicating that binding was protein-mediated and independent of SA (75). It remains to be explored how non-sialylated proteins bind to Siglecs, as the binding site and the mechanism of such binding is still unknown. This association activated the inhibitory signaling through the engagement of SHP1/2. Thus, suppressed host immune responses like oxidative burst, repressed phagocytic activity, and extracellular traps in the leucocytes, thereby favoring pathogen persistence (75).

Likewise, we had reported the presence of SA in *Pseudomonas aeruginosa* (PA) and identified a few sialoglycoproteins by mass spectrometry (80). These sialoglycoproteins interact with several human Siglecs *via* SA to enhance their pathogenicity (81–83). Interaction of *PA-*SA with inhibitory Siglec-9 on neutrophils reduced ROS production, elastase release, and decreased NETs formation, which altogether suppressed the activation of these immune cells (**Figure 1G**). Moreover, the association of the bacterial SA with murine Siglec-E on macrophages exhibited enhanced phagocytosis but reduced oxidative burst (**Figure 1H**) (83).

Our group also had demonstrated that *Leishmania donovani*, the causative agent of Indian visceral leishmaniasis displays different derivatives of sialic acid on its cell surface (84). Subsequently, it was also shown that various strains of *Leishmania* species causing different forms of the disease exhibited a differential distribution of SA on their surface. Binding studies of sialylated virulent *L.donovani* strain with soluble siglec-Fc chimeras displayed its high interaction only with Siglec-1 and Siglec-5. Furthermore, we also reported that these parasites interact with Siglec-E on murine macrophages to subvert the host immune response (85, 86). ROS production and other macrophage effector functions were upregulated by silencing Siglec-E, which ultimately diminished the parasite survival inside the host (83). This indicates that Siglec-E suppresses ROS production in parasite-infected macrophages (**Figure 1H**).

All this information supports the well-established immune-inhibitory role of Siglecs in the promotion of pathogen survival and disease progression. Thus, Siglecs at the host-pathogen interface can play a very important role in modulating the immune response through regulating ROS production in the immune cells.

## Discussion

The crucial role of Siglec-sialoglycan interactions in regulating immune and inflammatory responses is increasingly becoming relevant. Siglecs are being recognized as potential therapeutic targets in various inflammatory disorders and cancer  (87, 88). Strategies to target activatory or inhibitory Siglecs for regulating immune response involve modulation of overall sialylation, use of antibodies, or sialic acid mimetics (89–93).

Several studies demonstrate that Siglec-sialoglycan interactions additionally promote as well as inhibit ROS generation to control diverse cellular functions ranging from apoptosis to maintenance of cellular life-spans. Such interactions may be beneficial for modulating oxidative stress as a part of anti-tumor, anti-inflammatory therapy. Siglecs may induce immune-tolerance or suppress inflammation by depleting ROS-producing cells or inhibiting ROS release. A few Siglec agonists which enhanced Siglec-based suppression of ROS production in inflammatory disorders have already been demonstrated  (28, 42, 67–69).

However, the consequence of Siglec-sialoglycan interaction may vary depending upon the location of Siglec and the presence of its cognate ligand in the surroundings. Also, some of the Siglecs have redundant roles. More comprehensive studies are required to define the outcome of such signaling events before siglecs may be used as therapeutic agents. In this respect, the role of Siglec-sialoglycan interactions and protein-protein interactions in Siglec-based ROS regulation needs to be explored in detail.

## Author Contributions

All the authors enlisted have made substantial, direct, and intellectual contribution to the work and approved it in its final form.

## Conflict of Interest

The authors declare that the research was conducted in the absence of any commercial or financial relationships that could be construed as a potential conflict of interest.

## Publisher’s Note

All claims expressed in this article are solely those of the authors and do not necessarily represent those of their affiliated organizations, or those of the publisher, the editors and the reviewers. Any product that may be evaluated in this article, or claim that may be made by its manufacturer, is not guaranteed or endorsed by the publisher.
